# Effectiveness of antiviral metal and metal oxide thin-film coatings against human coronavirus 229E

**DOI:** 10.1063/5.0056138

**Published:** 2021-11-01

**Authors:** Louis-Vincent Delumeau, Hatameh Asgarimoghaddam, Tamiru Alkie, Alexander James Bryan Jones, Samantha Lum, Kissan Mistry, Marc G. Aucoin, Stephanie DeWitte-Orr, Kevin P. Musselman

**Affiliations:** 1 Department of Mechanical and Mechatronics Engineering, University of Waterloo, 200 University Ave. West, Waterloo, Ontario N2L 3G1, Canada; 2 Waterloo Institute for Nanotechnology, University of Waterloo, 200 University Ave. West, Waterloo, Ontario N2L 3G1, Canada; 3 Department of Health Sciences, Wilfrid Laurier University, 75 University Ave. West, Waterloo, Ontario N2L 3C5, Canada; 4 Department of Chemical Engineering, University of Waterloo, 200 University Ave. West, Waterloo, Ontario N2L 3G1, Canada

## Abstract

Virucidal thin-film coatings have the potential to inactivate pathogens on surfaces, preventing or slowing their spread. Six potential nanoscale antiviral coatings, Cu, Cu_2_O, Ag, ZnO, zinc tin oxide (ZTO), and TiO_2_, are deposited on glass, and their ability to inactivate the HCoV-229E human coronavirus is assessed using two methods. In one method, droplets containing HCoV-229E are deposited on thin-film coatings and then collected after various stages of desiccation. In the second method, the thin-film coatings are soaked in the virus supernatant for 24 h. The Cu and Cu_2_O coatings demonstrate clear virucidal behavior, and it is shown that controlled delamination and dissolution of the coating can enhance the virucidal effect. Cu is found to produce a faster and stronger virucidal effect than Cu_2_O in the droplet tests (3 log reduction in the viral titer after 1 h of exposure), which is attributed, in part, to the differences in film adhesion that result in delamination of the Cu film from the glass and accelerated dissolution in the droplet. Despite Ag, ZnO, and TiO_2_ being frequently cited antimicrobial materials, exposure to the Ag, ZnO, ZTO, and TiO_2_ coatings results in no discernible change to the infectivity of the coronavirus under the conditions tested. Thin-film Cu coatings are also applied to the polypropylene fabrics of N95 respirators, and droplet tests are performed. The Cu fabric coating reduces the infectivity of the virus; it results in a 1 order-of-magnitude reduction in the viral titer within 15 min with a 2 order-of-magnitude reduction after 1 h.

## I. INTRODUCTION

The SARS-CoV-2 virus spreads from symptomatic and pre/asymptomatic individuals,^1^ primarily through aerosols,^2–6^ but fomites^7–11^ and other modes of transmission are of concern.^12^ The surface stability of coronaviruses has been studied on surfaces in hospitals and homes,^7–9,13–20^ and SARS-CoV-2 has been repeatedly detected in samples taken from the surfaces of healthcare and public settings.^13–15,17^ Coronaviruses have shown persistence on surfaces, including those of personal protective equipment (PPE), for days under typical experimental conditions,^7–9,19^ although it is recognized that these conditions differ from those in real life.^21^ Encouragingly, specific surfaces such as copper and copper alloys have demonstrated the ability to inactivate these enveloped viruses.^8,9,22^

Comparatively little work has been done on engineered antiviral coatings for coronaviruses. In fact, the difficulties in assessing virucidal activity often hinder its examination.^23^ However, effective antiviral coatings could play an essential role in containing the spread of COVID-19 and future epidemics by inactivating viral particles before they have a chance to infect. Antiviral coatings have been reported previously for a range of viruses, including coatings based on metals (e.g., Ag, Cu, Au, and Fe), metal-oxide catalysts (e.g., TiO_2_, ZnO, and Fe_2_O_3_), nonmetallic materials (e.g., fullerenes and carbon nanotubes), and combinations thereof.^22,24–29^ There is a variety of ways in which the coatings can be antiviral through effects such as ion release, localized surface plasmon resonance, the generation of reactive oxygen species, and receptor inactivation.^30^ Micro- or nanostructured antiviral coatings may also take advantage of physical effects that are not present in bulk materials.^31–33^ The limited work on antiviral coatings can be contrasted with antibacterial coatings, which have received greater attention to date. Metal and metal-oxide coatings, sometimes combined with organic materials to form composite films, have repeatedly been shown to inhibit bacterial growth for a variety of bacteria through contact and noncontact mechanisms.^35–36^ The release of metal ions from the coatings was consistently found to be the main bactericidal mode of action^34,35^ with the extent of ion release correlated with antibacterial efficiency (at least in the case of Ag ions).^36^

Of the many possibilities, it remains to be seen what the most effective coatings are for inactivating coronaviruses. Coatings consisting of Cu_2_O nanoparticles bound with polyurethane,^37^ spray-coated Cu,^38,39^ spray-coated Cu-nanoparticle-containing resin,^40^ Cu nanowires coated with zeolitic imidazolate framework 8 (a porous metal–organic framework),^41^ anionic polymers,^42^ and TiO_2_ nanoparticles combined with ultraviolet-C radiation^43^ have thus far shown promise for the inactivation of coronaviruses.

In this work, we compared the ability of six potential nanoscale antiviral coatings to inactivate the HCoV-229E human coronavirus. Thin films of Cu, Cu_2_O, Ag, ZnO, zinc tin oxide (ZTO), and TiO_2_, almost all of which are frequently cited for their antimicrobial properties, were deposited on glass. HCoV-229E was then exposed to the thin-film coatings either in a droplet form to allow for partial to full desiccation or in a solution form with an excess medium to ensure no desiccation occurred. After incubation at room temperature, at specific time points, the remaining viable virus was quantified using median tissue culture infectious dose (TCID_50_) methods. Prior to virus exposure, the cytotoxicity of the coating materials to cells was quantified. The Cu coating was also deposited on nonwoven polypropylene fabrics used in N95 respirators, and the ability of the coated fabric to inactivate the coronavirus was assessed.

## II. MATERIALS AND METHODS

### A. Thin-film coatings

All glass and fabric substrates were first cleaned ultrasonically in ethanol for 10 min, then rinsed in deionized water, and dried with filtered air.

Cu and Ag films about 50 nm thick were coated on borosilicate glass substrates (70 × 70 × 1.1 mm^3^) or circular cover glasses (18 mm in diameter) by thermal evaporation. Cu pellets (99.999%, Angstrom Engineering) or Ag pellets (99.9%, Angstrom Engineering) were placed in an Angstrom–S-38B alumina-coated open boat, and the chamber was evacuated to 5 × 10^−6^ Torr. The deposited film’s thickness was controlled by a quartz crystal sensor. Some of the Cu films were converted to Cu_2_O by annealing at 225 °C for 30 min on a hot plate in air. The resulting Cu_2_O films were ∼71 nm thick, as evaluated by ellipsometry. Identical Cu depositions were performed on spunbond nonwoven polypropylene fabrics that are used as the outer (50 g/m^2^) and inner (25 g/m^2^) fabric layers in N95 respirators.

TiO_2_ films ∼40 nm thick were synthesized by spin coating on the same borosilicate glass substrates, which were first cut into pieces ∼10 × 10 mm^2^ in size. A 0.1M precursor solution was made by mixing titanium diisopropoxide and 1-butanol in a 2:41 volumetric ratio, and then the solution was filtered using a 0.45 *μ*m polytetrafluoroethylene (PTFE) filter. 50 *μ*l of the precursor solution was pipetted onto a glass substrate and spin coated at 4000 rpm for 10 s before being left to dry at 125 °C for 5 min. The spin coating and drying was repeated two more times so that three consecutive layers were applied. This was followed by a final annealing step at 450 °C for 30 min.

ZnO films about 135 nm thick were deposited by atmospheric-pressure spatial chemical vapor deposition (AP-SCVD) using a custom-built atmospheric-pressure spatial atomic layer deposition (AP-SALD) system. AP-SALD is a rapid, open-air version of atomic layer deposition. Recent review articles discuss the AP-SALD approach in detail.^44,45^ If the AP-SALD gas flows and the spacing between the AP-SALD reactor head and the substrate are selected to enable some mixing of the precursor and reactant in the gas phase, AP-SCVD can occur. AP-SCVD results in a higher film deposition rate while still producing conformal, pinhole-free films.^46^ Here, diethylzinc (DEZ, Fisher Scientific) was used as the precursor and deionized water was the reactant. 23 SCCM of N_2_ was bubbled through the DEZ bubbler, which was combined with a carrier N_2_ flow of 127 SCCM. 45 SCCM of N_2_ was bubbled through the water bubbler and combined with a carrier N_2_ flow of 255 SCCM. 150 SCCM of N_2_ was delivered to each inert gas curtain channel. The same 70 × 70 mm^2^ borosilicate glass substrates were heated to 80 °C, positioned ∼0.1 mm below the reactor head, and oscillated at 1.5 cm/s for 500 oscillations.

ZTO films ∼90 nm thick were also produced by AP-SCVD using DEZ, tetrakis(dimethylamino)tin (TDMASn, Strem Chemicals), and ozone. The TDMASn bubbler was heated to 80 °C. 100 SCCM of N_2_ was bubbled through the TDMASn and combined with a 60 SCCM carrier flow of N_2_. 45 SCCM of N_2_ was bubbled through the DEZ and combined with a 105 SCCM carrier flow of N_2_. 83 SCCM of N_2_ was delivered to each inert gas curtain channel. Approximately, 650 SCCM of ozone-oxygen mixture was delivered to the reactor head at a concentration of ∼280 g/Nm3 (grams of ozone per cubic meter at standard temperature and pressure, balance oxygen). The same 70 × 70 mm^2^ borosilicate glass substrates were held ∼0.2–0.3 mm below the reactor head, heated to 165 °C, and oscillated at 3 cm/s for 350 oscillations.

The larger (70 × 70 mm^2^) glass substrates and polypropylene fabrics were cut into pieces ∼10 × 10 mm^2^ in size, and all samples were stored in sterilized plastic boxes prior to virucidal analysis.

### B. Material characterization

The thicknesses of the Cu_2_O, TiO_2_, ZnO, and ZTO films were measured using a Film Sense FS-XY150 ellipsometer. All coatings were modeled using a Tauc–Lorentz model, and the ZnO and TiO_2_ coatings were also fit using a Cauchy model, which showed a good agreement with the thickness estimates obtained using the Tauc–Lorentz model. The measured thicknesses are presented in Table S1 of the supplementary material.

Grazing incidence x-ray diffraction (GIXRD) was performed from 2-theta values of 5°–80° at a scan rate of 0.02°/s with a PANalytical X’PERT PRO diffractometer system and Cu Kα radiation (wavelength of 1.5406 Å). X-ray photoelectron spectroscopy (XPS) was performed with a VG Scientific ESCALAB 250 XPS system and an Al Kα x-ray source. The C1s peaks were aligned to 284.6 eV to calibrate the spectra, and spectrum analysis was carried out with the CasaXPS software. Contact angle measurements were performed using 10 *μ*l droplets of deionized water. Two droplets were dispensed onto each coating, and a high-definition photo of each droplet was taken. The contact angle on each side of each droplet was measured using an ImageJ’s angle tool, and a mean contact angle was calculated for each material.

### C. Cells and virus used

MRC-5 embryonic lung fibroblast cells (ATCC^®^ CCL-171^TM^) and Hep G2 cells (ATCC HB-8065^TM^) were obtained from the American Type Culture Collection. Both cells were cultured in Eagle’s Minimum Essential Medium (EMEM) supplemented with 10% heat inactivated fetal bovine serum (FBS) and 1% penicillin/streptomycin and expanded in 75 cm^2^ tissue culture flasks (BD Falcon) to 80%–85% confluency. All cells were cultured at 37 °C in 5% CO_2_ in a humidified incubator, and FBS was inactivated at 56 °C for 30 min. The human coronavirus 229E (ATCC VR-740^TM^) was propagated in MRC-5 cells. Briefly, MRC-5 cells (75%–80% confluent) cultured in T-75 flasks were infected with human coronavirus 229E (HCoV-229E) in the serum-free EMEM for 2 h, and without removing the initial inoculum, the medium was supplemented with 2% FBS-containing EMEM for a final volume of 10 ml and then incubated at 33 °C for 3 days. The cells were freeze-thawed twice, harvested, and centrifuged at 10 000 × g for 10 min at 4 °C. The cell supernatant was collected, filtered through a syringe filter of 0.22 *μ*m pore size, aliquoted, and stored at −80 °C. The virus preparation used in this study had a titer of 4.48 × 10^4^ TCID_50_/ml, as determined by the Reed and Muench method.^47^

### D. Virucidal analysis of the coatings

To determine the virucidal efficacy of the coatings against the HCoV-229E strain, two methods, herein referred to as the droplet method (three independent trials each) and the wet method (two independent trials each), were used to expose the viruses to the antiviral coatings. In the droplet method, a 25 *μ*l droplet of HCoV-229E supernatant with a titer of 4.48 × 10^4^ TCID_50_/ml was added onto the surface of the various thin-film coatings on glass or respirator fabric. Uncoated glass substrates and respirator fabric were used as control materials and treated in the same way as the coated materials. The droplets were incubated at room temperature (20–22 °C), and the virus in the media from the surfaces of the glass substrates and fabrics was collected after 1 h (10% desiccation), 2.5 h (50% desiccation), 4.5 h (80% desiccation), and 7 h (100% or complete droplet desiccation). The medium was added to each volume collected for a total volume of 50 *μ*l and stored at −70 °C until virus titration. In addition, for some further tests on the Cu-coated fabrics, the supernatant was also collected after just 15 min (referred to as 0% desiccation) to observe the virucidal effect at shorter timescales. In the wet method, the coated and uncoated glass were placed into 12-well plates and soaked in 950 *μ*l of 4.48 × 10^4^ TCID_50_/ml virus supernatant for 24 h and the media were collected and stored at −70 °C until virus titration. The stock virus was back-titered in Hep G2 cells by taking 25 *μ*l of MRC-5-derived HCoV-229E, diluting it in 25 *μ*l of medium, and storing at −70 °C until titered on Hep G2.

All samples from the droplet-method and wet-method tests were fivefold serially diluted, and the diluted samples were added onto the Hep G2 cells that were cultured in 96-well microtiter plates and incubated at 33 °C for 7 days in 5% CO_2_ in a humidified incubator. Hep G2 cells were used as they were found to have a very low limit of detection of the virus at about 0.448 TCID_50_/ml. Indeed, Hep G2 cells are more permissive to HCoV-229E than MRC-5; as such, the MRC-5-derived stock titer was much lower than that derived on Hep G2 (stock virus, Figs. 4 and 6). The Hep G2 cells were observed under a microscope for cytopathic effects that indicated virus replication. The resulting virus titer was determined by the Reed and Muench method as indicated above.

Prior to exposure of HCoV-229E to the coatings, the cytotoxic effects of the coatings were measured. This test was performed to ensure that the cytotoxicity observed in the virus exposures was due to the virus replication that killed the cells, not the direct toxicity from the coated materials.^22^ The thin-film coatings were exposed to the plain media without the virus, and then the media were collected, serially diluted, and added to the Hep G2 cells. The cell viability was measured by incubating the cells with the fluorescence indicator dye alamarBlue (AB) and 5-carboxyfluorescein diacetate acetoxymethyl ester (CFDA-AM) (Invitrogen) for 1 h at 37 °C. Fluorescence was measured in a Synergy HT plate reader (BioTek, Winooski, VT) at the excitation/emission wavelengths of 530/590 and 485/528 nm for AB and CFDA-AM, respectively.

### E. Statistical analysis

Statistical analysis was carried out with the statistical programming language R (version 4.1.1)^48^ using the aov function [Fit an Analysis of Variance (ANOVA) Function]. Briefly, models were developed to assess main effects and multifactor interactions on virus titers (log transformed), and the Akaike information criterion,^49^ as part of the AICcmodavg library package,^50^ was used to assess the best fit. Tukey’s honestly significant difference post hoc test was used to analyze and compare mean viral titers between conditions.

## III. RESULTS AND DISCUSSION

### A. Coating characterization

**FIG. 1.**
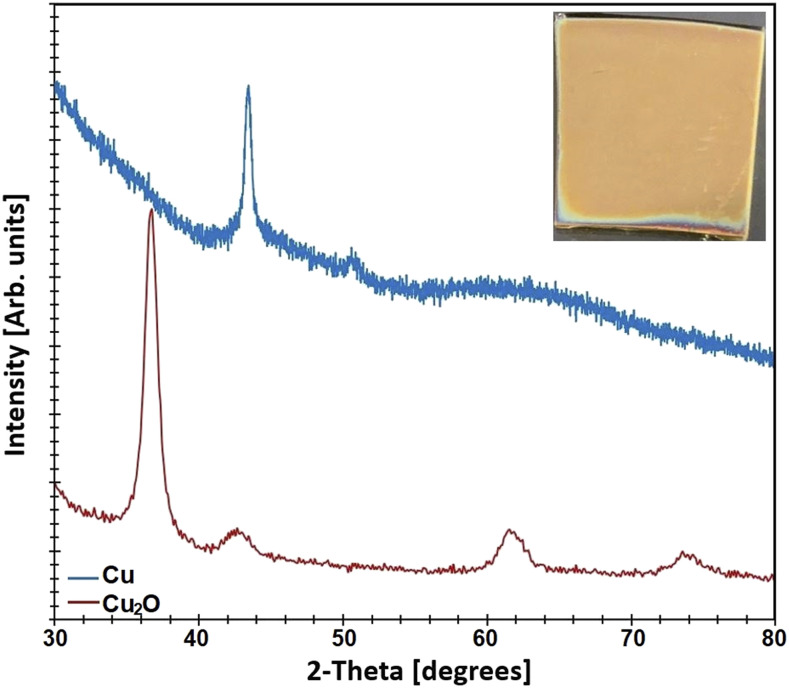
GIXRD data for a Cu_2_O and Cu thin-film coating on the glass. Inset: picture of a Cu_2_O film.

GIXRD was used to confirm the formation of Cu_2_O coatings via thermal oxidation of evaporated Cu. Figure 1 shows the GIXRD data for one of the Cu_2_O coatings on borosilicate glass. The peaks visible at 37°, 43°, 62°, and 74° correspond to the (111), (200), (220), and (311) planes of Cu_2_O, respectively.^33,51,52^ CuO peaks at 36° and 39° were not observed.^53^ The GIXRD data for a Cu coating are also provided for reference in Fig. 1. The peaks at about 43.5° and 50.5° correspond to the (111) and (200) planes of copper, respectively.^53^

**FIG. 2.**
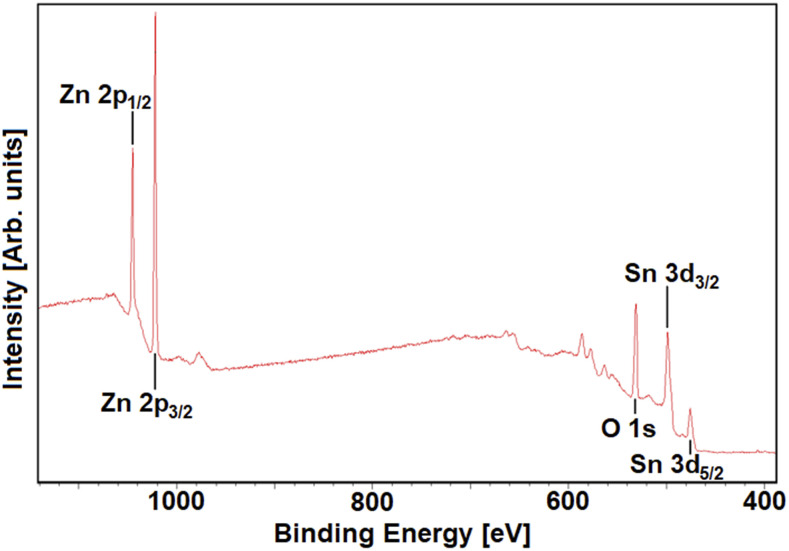
XPS spectrum for a ZTO coating on the glass.

The properties of the ZnO films produced by AP-SCVD and the TiO_2_ films produced by spin coating have been reported previously.^54–56^ XPS was used to confirm the deposition of the ZTO films on borosilicate glass by AP-SCVD. An XPS spectrum is shown in Fig. 2, where the expected peaks for Zn, Sn, and O are seen.^57,58^ Analysis of the spectrum indicated a Sn:Zn ratio of ∼2:5. The measurement was repeated for different ZTO films, which indicated a uniform composition across the samples.

The contact angles of a water droplet on the Ag, Cu, Cu_2_O, TiO_2_, ZnO, and ZTO coatings on the glass were measured to be 75° ± 1°, 90° ± 3°, 85° ± 1°, 56° ± 2°, 65° ± 1°, and 75° ± 4°, respectively, indicating that all coatings, except for Cu, were slightly hydrophilic (images provided in Table S2 of the supplementary material).

### B. Virucidal performance

**FIG. 3.**
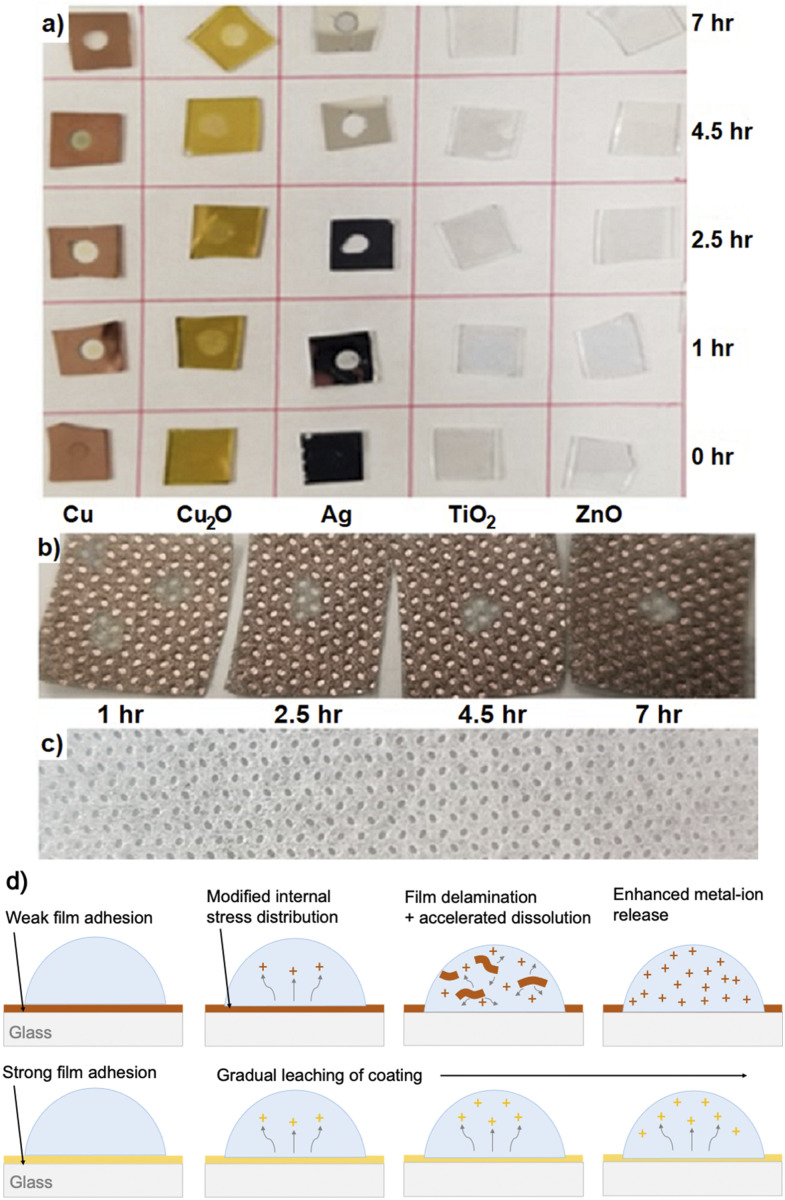
(a) Coatings on the glass after a droplet test. Removal of the Cu and Ag coatings from the glass substrate during the test is evident. (b) Picture of Cu coatings on the respirator outer fabric after a droplet test. Cu removal takes longer than on the glass. (c) Uncoated outer fabric for comparison. (d) Ion release from a delaminated (upper) and adhered (lower) coating.

Coated glass and outer-fabric samples from the droplet-method tests are shown in Figs. 3(a) and 3(b), respectively. Droplet exposure resulted in delamination and dissolution of some of the thin-film coatings, particularly the Cu and Ag deposited on the glass by evaporation. Delamination of thin films is known to occur due to the presence of internal stresses, which are concentrated at edges or flaws in the material.^59^ Removal of the Cu and Ag films from the glass was observed to be almost complete following 1 h of exposure to the droplet [Fig. 3(a)]. The delamination may be attributable to leaching of metal at the droplet-coating interface and/or interaction forces at this interface, both of which would influence the stress distribution in the coating. No Cu or Ag flakes were observed when collecting the media from the Cu and Ag samples, nor were Cu or Ag particles visible during the microscopy analysis, indicating that the Cu and Ag films were dissolved in the droplets. While the release rate of metal ions is known to vary with factors such as the composition and volume of the media,^60^ recent work from one of the authors measured leached Cu-ion concentrations greater than 10 mM in virus droplet tests,^22^ which are higher than the concentration that would result from complete dissolution of the 50 nm Cu film in the 25 *μ*l droplet.

The Cu coating was leached from the outer fabric gradually, as shown in Fig. 3(b) [uncoated fabric is shown in Fig. 3(c) for reference]. This is attributed to the fact that the porous fabric surface is expected to result in a smaller contact area with the droplet and different film adhesion properties. Similarly, rapid delamination of the Cu_2_O coatings on the glass was not observed [see Fig. 3(a)], presumably due to superior adhesion to the glass substrate imparted by the thermal oxidation process. Gradual leaching of the Cu_2_O coating is instead shown in Fig. 3(a). The faster dissolution of the Cu and Ag coatings on the glass is likely due to the observed film delamination, which results in suspension and break-up of the films in the droplet, providing a much larger surface area for leaching of metal ions, as illustrated in the upper panel of Fig. 3(d). This is compared to the gradual leaching of a well-adhered Cu_2_O film in the lower panel of Fig. 3(d). It is important to note that metal incorporation into the media did not result in any apparent cytotoxic effects to the Hep G2 cells. Virus-free controls were added to the Hep G2 cells, and no cytotoxicity was measured (data not shown).

**FIG. 4.**
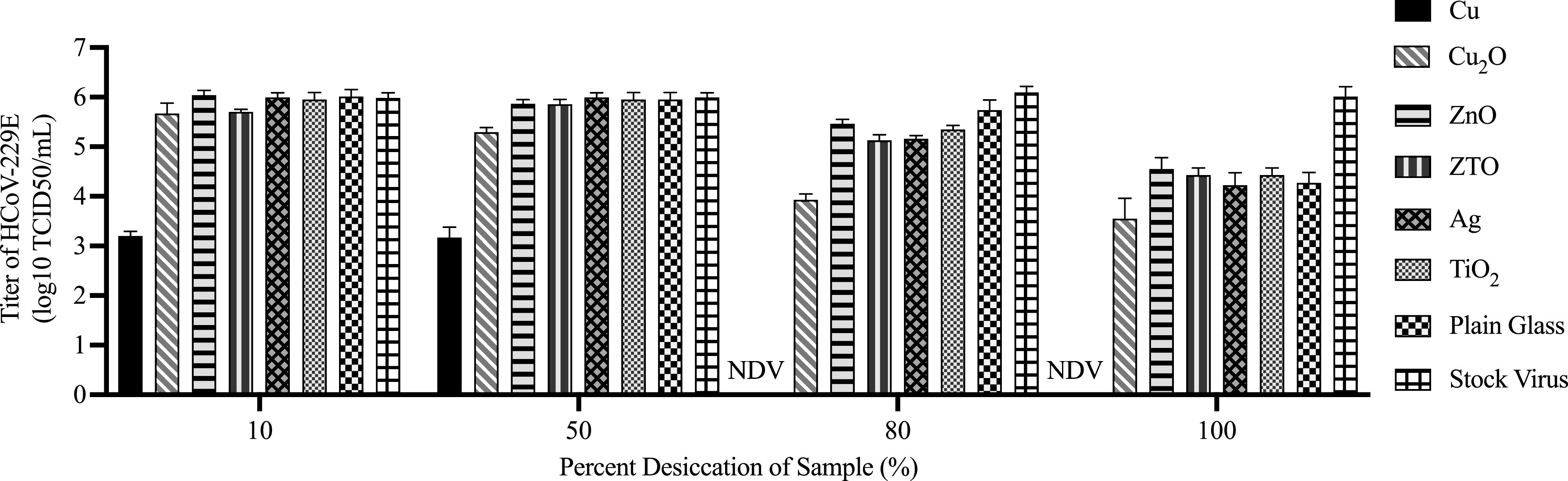
Virucidal properties of various thin-film coatings on the borosilicate glass. A droplet of HCoV-229E added on a coating was collected at various stages of desiccation on the glass. When droplets were completely desiccated, the cell culture media were added step by step to the desiccated spots to collect the entire contents. Viral titration was conducted on the Hep G2 cells following a standard protocol. The viral titer results are from three trials, and the bar graphs indicate the mean ± standard error of the mean. NDV indicates no detectable virus.

Figure 4 shows the viral titers for the droplet-method tests on the glass, where droplets containing the HCoV-229E virus were exposed to the different thin-film coatings for different lengths of time (expressed as a percentage desiccation). Viral titers for droplets exposed to plain borosilicate glass, as well as the stock virus, are also shown for comparison. The raw data are provided in Table S3 of the supplementary material. The relationship between the coating type and the desiccation level on the virus titer was assessed using ANOVA. AIC model selection was used to distinguish among a set of possible models describing the relationship between coatings and desiccation levels. The best-fit model, carrying 100% of the cumulative model weight, included an interaction effect between the coating type and the desiccation level, implying that the size of the effect on the titer due to the coatings is dependent on the desiccation level (see Table S4 of the supplementary material).

The non-copper-containing metals and metal oxides, namely, ZnO, ZTO, Ag, and TiO_2_, did not show viral titers that were significantly lower than those of the droplets left on plain glass at any stage of the desiccation studied (Table S4). This contrasts with the frequently reported antimicrobial properties of these materials and suggests that they may not be effective as antiviral coatings for coronaviruses under the experimental conditions tested. It should be recognized that these coatings may demonstrate virucidal properties under different conditions. For example, TiO_2_ and ZnO are photocatalytic and so might demonstrate a virucidal effect with exposure to UV light, and Ag may be more effective in the nanoparticle form where the nanoparticles can directly bind to viral proteins.^24,29^ Nonetheless, these results indicate that thin films of ZnO, ZTO, Ag, and TiO_2_ do not inactivate the HCoV-229E under ambient laboratory conditions.

In contrast, the copper-containing thin films demonstrated strong virucidal effects. At 10% droplet desiccation (1 h exposure to the coatings), the titer for Cu on the glass was 3 orders of magnitude below the other materials. The viral titer for the Cu_2_O coating, however, was more similar to that of the plain glass and stock solution. At 50% desiccation, the titer for the Cu coating was similar, but the titer for the Cu_2_O coating was now about 1 order below that of the plain glass and the stock solution. At 80% desiccation, some decrease in the viral titer was seen for all materials relative to the stock virus, but it was still only the copper-containing coatings that showed a significant decrease relative to the plain glass titer. The titer for Cu_2_O was now more than 1 order below that of the plain glass, while the Cu-coated glass sample had no discernible infectious potential left (a full 5–6 orders below the other materials). At 100% desiccation of the droplets (7 h exposure), all the coatings (including ZnO, ZTO, Ag, and TiO_2_) and the plain glass displayed significant reductions in the virus titer relative to the stock virus, which may be related to gradual inactivation of the virus in the medium, as well as physical desiccation of the droplet that may compromise the virus, or more specifically the virus envelope. Virus inactivation in water droplets has been attributed to the increasing concentration of solutes in water droplets as they dry, which can bring about a drop in pH and conditions that lower virus viability.^61–63^ It has been reported that the HCoV-229E virus may have a shorter survival time on surfaces than SARS-CoV, a genetically similar virus to SARS-CoV-2.^18,64^ It was still only the Cu and Cu_2_O coatings that showed an enhancement relative to the plain glass. Cu_2_O demonstrated a viral titer about 1 order below the plain glass, and the Cu on the glass still had no detectable infectious potential.

**FIG. 5.**
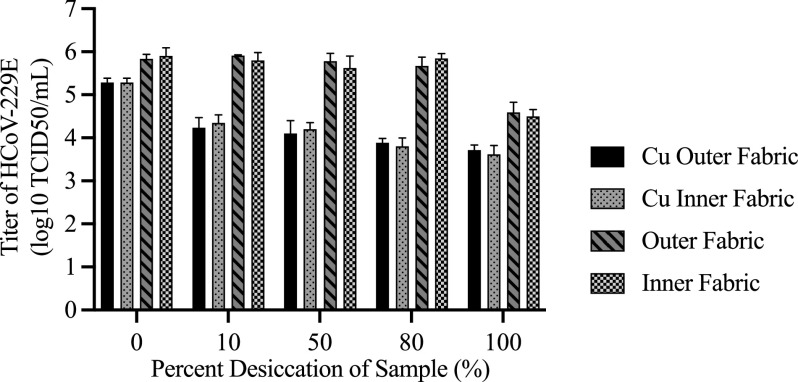
Virucidal effects of Cu coatings on the outer and inner polypropylene fabrics of N95 respirators. A droplet of HCoV-229E added on a coating was collected at various stages of desiccation on the fabric. When droplets were completely desiccated, the cell culture media were added step by step to the desiccated spots to collect the entire contents. Viral titration was conducted on the Hep G2 cells following standard protocols. The viral titer results are from three trials, and the bar graphs indicate the mean ± standard error of the mean.

Following the clear virucidal properties of the Cu-containing films on the glass, the droplet-method tests were carried out on Cu-coated polypropylene fabrics used in N95 respirators. Figure 5 shows the results comparing the viral titers of droplets exposed to Cu-coated and uncoated polypropylene fabrics (inner and outer) at different degrees of droplet desiccation. Here, an additional measurement at 0% desiccation, corresponding to a 15 min exposure, was introduced to test the ability of the coated fabrics to rapidly inactivate the coronavirus. The raw data are provided in Table S5 of the supplementary material. To probe the relationship between the coating, fabric type, and desiccation level on the virus titer, ANOVA was performed. AIC model selection was used to distinguish among a set of possible models. The best-fit model, carrying 100% of the cumulative model weight, included an interaction effect between the material being coated with Cu and the desiccation level. The type of fabric (inner and outer) did not play a role in explaining any variability in the data. In fact, there was no statistical significance found between the viral titers measured after exposure on inner and outer material (see Table S6 of the supplementary material).

Up to and including 80% desiccation, the droplets in contact with the uncoated fabrics experience no notable drop in the viral titer. Even after 15 min of exposure, the titers for the Cu-coated fabrics are about an order below their uncoated counterparts. This trend persists and increases to about 2 orders at the later desiccation stages. It is by 10% desiccation (1 h) that most of the decrease in infectious potential is achieved by the Cu coatings, showing a rapid effect. After complete drying of the droplets, the difference between the viral titers of the coated and uncoated fabrics is less marked since the titers of the droplets on the uncoated fabrics have decreased, but the difference remains significant (Table S6): about 1 order of magnitude.

It is noted that the virucidal effect of the Cu coating was not as large or rapid on the respirator fabrics (Fig. 5) as on the glass (Fig. 4). This may be attributable to the fact that the evaporated Cu thin-film coating on the glass was delaminated and dissolved more quickly than the coating on the fabrics, as shown in Figs. 3(a) and 3(b). For the droplet test on the Cu-coated glass, this would have resulted in the incorporation of a significant amount of Cu (∼4 *μ*g based on the film thickness and droplet area) into the 25 *μ*l droplet and the collected medium, increasing the interaction between the Cu ions and the virus. Furthermore, the concentration of the Cu ions in the droplet would be expected to increase as the desiccation of the droplet progressed. Notably, a significant reduction in viral titer was observed for the Cu-coated glass in Fig. 4 between 2.5 h (50% desiccation) and 4.5 h (80% desiccation), even though the coating was completely delaminated and dissolved after ∼1 h, which can be attributed to the increased concentration of Cu ions in the smaller droplet.

It has been shown that the antibacterial efficiency of Cu and Cu-alloy surfaces increases with the release rate of Cu ions from the surface,^65^ and recently, the concentrations of leached metals, including copper, from a surface were directly compared to the virucidal activity with an enveloped baculovirus.^22^ For the Cu-coated glass studied here, the entire Cu film was released into the droplet within 1 h. Less Cu would have been incorporated into the droplets and collected media on the fabrics. This highlights that the adhesion of an antiviral coating to the substrate may impact both the durability of the coating and its virucidal effect on incident virus-containing droplets via enhanced metal ion release, as illustrated in Fig. 3(d). Depending on the substrate and application, an appropriate coating method can be selected. For example, in contrast to evaporation that provides weak film adhesion, AP-SCVD results in the formation of chemical bonds with the underlying substrate such that stronger adhesion is expected.

**FIG. 6.**
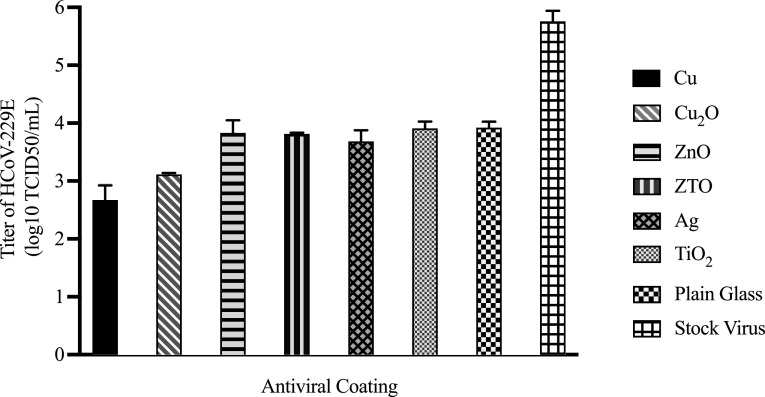
Virucidal properties of various thin-film coatings on the borosilicate glass. The coatings were completely soaked (wet method) in HCoV-229E-containing supernatant for 24 h. The supernatant was collected, and viral titration was conducted on the Hep G2 cells following a standard protocol. The viral titer results are from two trials, and the bar graphs indicate the mean ± standard error of the mean.

Figure 6 shows the viral titers for the wet-method tests, where the coated glasses were soaked in the virus supernatant for 24 h before the media were collected for virus titration (raw data in Table S7 of the supplementary material). As in the droplet tests, the viral titers for the ZnO, ZTO, Ag, and TiO_2_ exposures were approximately the same as the viral titer for the plain glass (Table S8), suggesting that these coatings do not enhance the virucidal properties of the glass under the testing conditions considered. Again, the reductions compared to the stock virus are attributed to the fact that HCoV-229E is not stable in the medium for extended periods under ambient conditions. The Cu- and Cu_2_O-coated glasses were more successful, showing titers about 1 order of magnitude lower. These reductions are smaller than those observed in the droplet-method tests in Fig. 4. This is likely due to the larger volume of viral solution used in the wet-method test (950 *μ*l), which would result in a smaller Cu-ion concentration. It is also noted that the virucidal effects of the Cu- and Cu_2_O-coated glasses are more similar in Fig. 6 than in the droplet-method tests. This may again be attributable to the varying degrees of coating delamination and dissolution. As noted earlier, the Cu_2_O coatings on the glass were not removed to the same extent as the Cu and Ag in the droplet-method tests [see Fig. 3(a)]. As a result, fewer Cu ions from the Cu_2_O coating (as compared to the Cu coating) would have been incorporated into the droplets to interact with the virus. In the wet-method tests, gradual fading was observed for all coatings, indicating gradual leaching. The absence of film delamination for the Cu and Ag coatings in the wet-method tests likely follows from the fact that the coatings were completely covered by the virus supernatant, preventing uneven stress distributions within the films. The more similar leaching rates, combined with the longer (24 h) exposure time that provided more time for leaching of the different coatings, are expected to have resulted in more similar Cu-ion concentrations, resulting in more similar responses for Cu_2_O and Cu in Fig. 6.

The results observed here are consistent with copper’s well-established capability as an antimicrobial material.^23,66–69^ Different explanations have been put forward to explain the antiviral or antibacterial effect of material surfaces, including the release of metal ions or reactive oxygen species, as well as direct contact between the material and pathogen.^67,69–72^ After testing these mechanisms of antiviral activity using bacteriophage Qβ, Sunada *et al.* concluded that it was mostly contact with the surface of Cu_2_O that was responsible and therefore a direct contact effect.^71^ Similar antiviral effects from direct contact have also been reported for the SARS-CoV-2 virus on CuO.^70^ In this work, the enhanced antiviral response observed for Cu coatings that rapidly delaminated and dissolved in virus-containing droplets suggests that Cu-ion release is the dominant mechanism for killing HCoV-229E, consistent with previous reports for some other viruses.^22,67,71^ The relative effect of these antiviral mechanisms likely varies for different coating compositions, surface features, and pathogens. Furthermore, pathogens may be harmed in several ways simultaneously by a Cu-containing coating, including nucleic acid denaturation, protein activity inhibition, damage to the virus capsid, and plasma membrane permeabilization.^23,66,67,69^ The viral RNA genome was shown to be destroyed by Cu for the case of a norovirus, and the material also lowered the copy number of a gene encoding a viral protein playing a crucial role in infection.^68^ Further research into the antiviral mechanisms for engineered Cu-containing coatings and coronaviruses is warranted.

## IV. CONCLUSION

Six potential nanoscale antiviral coatings—Cu, Cu_2_O, Ag, ZnO, ZTO, and TiO_2_—were tested for their ability to inactivate the human coronavirus HCoV-229E. The Cu and Cu_2_O thin-film coatings demonstrated strong virucidal effects. The ZnO, TiO_2_, Ag, and ZTO coatings (the latter tested for antiviral properties for the first time here) were not found to decrease the infectivity of the coronavirus as compared to a plain glass surface. However, it is possible that these coating materials could display virucidal behavior under different testing conditions.

The Cu coatings were found to provide the fastest and strongest virucidal effect in tests with 25 *μ*l droplets (2–3 log reduction in the viral titer after 1 h of exposure on the glass). This was attributed, in part, to the delamination of the Cu coatings and their dissolution into the droplets, which would have increased the Cu ion–virus interaction in the droplets and collected media (enhanced metal ion release). In contrast, the Cu_2_O coatings showed better adhesion to the glass substrates. A more similar virucidal effect was observed for the Cu and Cu_2_O coatings in the wet method, where delamination of the Cu coating was not observed, and the coatings interacted with a larger volume of supernatant for a longer period. These results suggest that the control of film adhesion can be leveraged to tune the virucidal behavior of engineered coatings. A loosely adhered Cu coating, for example, could be useful in a PPE application where rapid virus inactivation in an aqueous medium is key (if applied safely), whereas a more adhesive Cu or Cu_2_O film that remains intact on the substrate could be useful for applications such as subway handrails to have a lasting effect. Further studies on the delamination, dissolution, and virucidal efficacy of engineered coatings as a function of the droplet size are warranted. The relationship between the coating properties and the inactivation of coronaviruses also warrants investigation, as the properties of Cu coatings, such as roughness and grain structure, have been shown to influence the antimicrobial response for other pathogens.^73,74^

Analysis of Cu-coated N95 respirator fabrics indicated that the coatings reduced the infectivity of the virus by 1 order of magnitude within 15 min and resulted in a 2 order reduction of the viral titer after 1 h. This is promising for inactivating coronaviruses on contaminated PPE to protect wearers and limit the spread of the pathogen.

## SUPPLEMENTARY MATERIAL

See the supplementary material for ellipsometry-determined coating thicknesses, contact angle measurements, viral titer data, and statistical analysis.

## Data Availability

The data that support the findings of this study are available within the article and its supplementary material.
